# A Preliminary Study on Change of Serum Immunoglobulin G Glycosylation in Patients With Migraine

**DOI:** 10.3389/fneur.2022.860555

**Published:** 2022-05-23

**Authors:** Jingwei Xu, Yuan Wang, Yating Han, Ningfeng Liu, Zhenming Liu, Huailian Guo, Xiajuan Zou, Jun Zhang

**Affiliations:** ^1^Department of Neurology, People's Hospital, Peking University, Beijing, China; ^2^Department of Neurology, The First Medical Center of PLA General Hospital, Beijing, China; ^3^State Key Laboratory of Natural and Biomimetic Drugs, School of Pharmaceutical Sciences, Peking University, Beijing, China; ^4^Beijing Key Laboratory of Tumor Systems Biology, Medical and Healthy Analysis Center, Peking University, Beijing, China; ^5^Key Laboratory for Neuroscience, Ministry of Education, National Health and Family Planning Commission, Peking University, Beijing, China

**Keywords:** migraine, IgG N-glycosylation, N-glycopeptide, MALDI-TOF-MS, machine learning

## Abstract

**Background and Objective:**

Migraine is a common neurological disease, but its pathogenesis is still unclear. Previous studies suggested that migraine was related to immunoglobulin G (IgG). We intended to analyze the immune characteristics of migraine from the perspective of IgG glycosylation and provide theoretical assistance for exploring its pathogenesis.

**Methods:**

The differences in the serum level of IgG glycosylation and glycopeptides between patients with episodic migraine and healthy controls were analyzed by applying the poly(glycerol methacrylate)@chitosan (PGMA@CS) nanomaterial in combination with matrix-assisted laser desorption/ionization time-of-flight mass spectrometry (MALDI-TOF-MS). We constructed a binary classification model with a feedforward neural network using PyTorch 1.6.0 in Python 3.8.3 to classify the episodic migraine and healthy control groups.

**Results:**

Twenty patients with migraine and 20 healthy controls were enrolled and the blood samples and clinical information were collected. Forty-nine IgG N-glycopeptides were detected in the serum of the subjects. The serum level of N-glycopeptide IgG1 G0-NF (*p* = 0.012) was increased in patients with migraine. The serum level of N-glycopeptide IgG3/4 G2FS (*p* = 0.041) was decreased in patients with migraine with family history of headache. It was found that the serum level of the IgG1 G1 (*p* = 0.004) and IgG2 G0 (*p* = 0.045) was increased in patients with migraine with aura, while the serum level of IgG2 G0N (*p* = 0.043) in patients with migraine with aura was significantly lower than that in patients with migraine without aura. In addition, a linear feedforward neural network (FFNN) was used to construct a binary classification model by detected IgG N-glycopeptides. The area under the curve (AUC) value of the binary classification model, which was constructed with 7 IgG N-glycopeptides, was 0.857, suggesting a good prediction performance. Among these IgG N-glycopeptides that were constructed the model, IgG1 G0-NF was overlapped with the differential IgG N-glycopeptide between patients with migraine and healthy controls detected with MALDI-TOF-MS.

**Conclusion:**

Our results indicated that the serum level of N-glycopeptides IgG1 G0-NF might be one of the important biomarkers for the diagnosis of migraine. To the best of our knowledge, this is the first study about the changes of IgG N-glycosylation in patients with migraine by the method of MALDI-TOF-MS. The results indicated a relationship between the migraine and immune response.

## Introduction

Migraine is a primary headache, characterized by recurrent pulsating moderate or severe headache, which is typically unilateral but sometimes bilateral. It is often accompanied by nausea, vomiting, photophobia, and phonophobia. About one-third of patients have visual, somatosensory, or other kinds of aura (1). The prevalence of migraine is 14.7% globally and 9.3% in China (2). Migraine is considered to be the seventh most disabling disease in the world, seriously affecting the quality of life of patients (3). Several hypotheses have been proposed about the mechanism of migraine, such as trigeminal nerve vascular theory (4) and cortical spreading depression theory (5). However, these theories still cannot fully explain the pathogenesis of migraine. Currently, the diagnosis of migraine mainly depends on the symptoms of patients, lacking specific laboratory biomarkers.

Although the exact pathophysiology has not yet been determined, it has been reported that restricting the intake of sensitized or intolerant food in patients with migraine complicated with irritable bowel syndrome (IBS) can reduce the frequency of migraine and abdominal pain (6). Aydinlar et al. proposed that the inflammatory response caused by the increase of immunoglobulin G (IgG) antibodies played a certain role in migraine attacks (7). Li et al. and Lu et al. reported that the IgG content of patients with migraine was significantly higher than that of the control group (8, 9) and negatively correlated with the ictal phase (8). It was long thought that the effects of calcitonin gene-related peptide and substance P release from peripheral terminals only have correlation with vasodilation and increased capillary permeability (10, 11). However, the nervous system also has the potential means to modify immune function by the neuropeptide-mediated signaling from sensory neurons to immune cells (12–14). Michoud et al. revealed a link between nociceptors and immune cells in 2020 (15). All of these findings suggest that there is a certain correlation between migraine and IgG antibodies.

Immunoglobulin G is the core component of antibodies and an important serum glycoprotein with glycosylation modification. The fragment crystallizable (Fc) N-glycans of IgG undergoes specific changes in abnormal physiological or pathological conditions (16). It has been found that the sialic acid residue reduced in rheumatoid arthritis and immune-mediated osteoporosis (17, 18), the galactose and N-acetylglucosamine decreased in osteoarthritis (19), and the core fucose increased in liver cancer (20). Therefore, the detection of IgG N-glycosylation variation can be used to monitor the immune status, thereby assisting the early diagnosis and prognosis assessment of related diseases. However, the studies on IgG N-glycosylation were mainly focused on immune and tumor diseases. Currently, with the maturity of detection technology, studies have been conducted on nonimmune and nonneoplastic diseases. Freidin et al. investigated the correlation between IgG N-glycosylation and lower back pain and found multiple-related glycan modules (21). Lundström et al. found that the fucosylation of IgG1 increased and the levels of galactose and N-acetylneuraminic acid decreased in patients with Alzheimer's disease (AD) (22). The relationship between the glycosylation and diseases is attracting an increasing attention, but the change of serum IgG glycosylation in patients with migraine has not been reported.

Mass spectrometry (MS)-based glycoproteomics is a high-throughput and powerful approach for system-wide screening of glycosylation-based biomarkers (23). It has already been demonstrated that the use of MS can investigate aberrations in glycosylation associated with several diseases (24). In this study, the serum IgG N-glycosylation profile and N-glycopeptides were studied in patients with migraine and healthy controls by MS and the difference between the two groups was compared. Meanwhile, the difference in IgG glycosylation between subgroups of migraine, including the different subtypes, phases, and family history, was also analyzed. This study aims to explore the potential pathogenesis and biological markers of migraine.

## Materials and Methods

### Study Cohort

In this study, 40 subjects were enrolled, including 20 patients with migraine (episodic migraine) and 20 healthy individuals (Table 1). The baseline data was collected to determine the subtypes of migraine (with or without aura) and whether the patients had a family history of headache. The phases of migraine (ictal or interictal) were inquired when the blood samples were collected. Patients with migraine who met the migraine diagnostic criteria of the International Classification of Headache Disorders 3rd Edition (ICHD-3) (1) and had a headache history of more than 1 year were selected as patients with migraine. The enrolled healthy individuals were age and sex matched and did not have any headache history. In addition, the subjects with the following characteristic were excluded: (1) Patients with severe infection, blood system disease, liver disease, malignant tumor, severe mental disease, or immune system disease; (2) Subjects who have received blood product transfusions in the past 6 months; and (3) Pregnant and lactating women.

**Table 1:** Characteristics of patients with migraine and controls.

	**Migraine group**	**Control group**	**t/χ^2^**	***P*** **value**
	**(*n* = 20)**	**(*n* = 20)**		
Gender	7/13	9/11	0.417	0.519
(male/female)				
Age(years)	40.1 ± 11.2	39.5 ± 11.0	0.194	0.847
(mean±SD)				
BMI(kg/m^2^)	22.15 ± 2.03	24.58 ± 3.05	−2.911	0.006^*^
(mean±SD)				
Ictal phase	7 (35%)	/	/	/
Migraine with aura	5 (25%)	/	/	/
Family history of headache	11 (55%)	/	/	/
Comorbidities		/	/	/
Hypertension	1 (5%)	1 (5%)	0.000	1.000
Hyperlipidemia	2 (10%)	2 (10%)	0.000	1.000
Allergic diseases	9 (45%)	3 (15%)	4.286	0.038^*^

*Statistical method: Student's t-test, chi-square test;*

* *p < 0.05*.

This study was approved by the Medical Ethics Committee of the Second Clinical Hospital of Peking University Health Science Center. All the subjects provided a written informed consent before this study.

### Blood Sample Collection and Serum Preparation

Blood samples (4 ml) were taken from the antecubital vein in citrated serum tubes (BD Biosciences), after an overnight fast. After centrifuged at 2,000–3,000 g for 10 min, the supernatant was collected into a test tube and the protease inhibitors were added at a ratio of 1:500. The mixture was aliquoted into 200 μl and stored in a refrigerator at −80°C.

### Immunoglobulin G Isolation and Digestion

The isolation of IgG from human serum was the same as the previous study (25) with protein G beads (Kirgen Biosciences, Shanghai, China). After measuring the concentration of protein IgG from human serum by a bicinchoninic acid (BCA) protein assay, the IgG was treated with a tube-gel digestion.

Immunoglobulin G proteins (10.0 μg) were heated at 95°C for 15 min for degeneration. After recovering to room temperature, the samples were mixed with 10.0 μl acrylamide/bis solution (30%, w/v; Bio-Rad, Hercules, California, USA), 6.5 μl Tris-HCl solution (1.5 M, pH 8.8; Macgene), 0.5 μl sodium dodecyl sulfate (SDS) solution (10%, w/v; Beijing Biotechnology Corporation Ltd.), and 2.0 μl ammonium persulfate solution (10%, w/v; Bioss Antibodies, Woburn, Massachusetts, USA). Finally, 1.0 μl N,N,N',N'-tetramethylethylenediamine (TEMED) (Bioss Antibodies) were mixed immediately and the polymerization reaction was conducted for at least 30 min at room temperature.

After the gel was formed in the tube, it was washed with ddH_2_O for 2 h. Then, the gel pieces were washed and dehydrated successively with acetonitrile (ACN) (Thermo Fisher Scientific) and 100 mM NH_4_HCO_3_/ACN (1:1, v/v). Proteolytic digestion was performed with trypsin (1:50, mass ratio) dissolved in NH_4_HCO_3_ (50 mM) at 37°C overnight. After digestion, gel pieces were eluted with 5% formic acid/ACN (1:2, v/v). Finally, the digested peptides were vacuum-dried.

### Enrichment of N-Glycopeptides

N-glycopeptides were enriched from the digested samples with poly(glycerol methacrylate)@chitosan (PGMA@CS) nanomaterial according to the previous report by Jie et al. (26). Briefly, digested samples were redissolved with loading buffer [89% ACN-3% trifluoroacetic acid (TFA) solution, v/v] and added to PGMA@CS incubating on an Eppendorf shaker. After incubation, PGMA@CS was washed with 89% ACN-3% TFA solution twice times and dehydrated with ACN. Finally, IgG N-glycopeptides were eluted with 30 μl of 20 mg/ml dihydroxybenzoic acid solution (70% ACN−1% H_3_PO_4_, v/v) for 20 min at 30°C.

### Matrix-Assisted Laser Desorption/Ionization Time-Of-Flight Mass Spectrometry Analysis

A volume of 0.6 μl prepared sample was loaded onto 384 polished stainless steel matrix-assisted laser desorption/ionization (MALDI) target plates. MALDI-time-of-flight (MALDI-TOF) mass spectra were acquired on the AXIMA-CFP Plus Mass Spectrometer (Kratos Analytical Ltd., Shimadzu Corporation, Kyoto, Japan), equipped with a nitrogen laser (337.1 nm). Mass spectra were obtained in the positive ion and linear mode with an acceleration voltage of 20 kV. The power was set to 105 kV and the ions between m/z 1,000 and 4,500 were acquired. Each mass spectrum was automatically generated by averaging 200 laser shots and the optimal acquisition point was m/z 2,602. Each spectrum was internally calibrated with the theoretical mass of the IgG N-glycopeptides (m/z 2602.0, 2796.1, 2958.2, and 3217.3).

### Data Extraction and Statistical Analysis

Mass spectrometry (MS) data were processed using Launchpad V2.4 Kompact MALDI software with a threshold of 0.050 mV and a top-hat baseline subtraction. The peaks between m/z 2,450 and 3,500 cm^−1^ were exported for statistical analysis. Missing values were replaced by zero. We used the ratio value of each N-glycopeptide peak height to the sum of heights of all the Fc N-glycopeptides in the same profile as its relative intensity. Four parallel experiments were performed for each sample and the average value was the final percentage. N-glycopeptides intensities between patients and controls were compared with the two-tailed Student's *t*-test in IBM SPSS statistics version 26 and the box plots were made by Origin 2021.

Given that many of these structures have the same glycan features such as bisecting GlcNAc, fucosylation, and galactosylation, which are closely related to IgG activity, additional derived glycan traits were calculated according to the following formulas: the abundance of bisecting GlcNAc (bi-N): G0N + G0NF + G1N + G1NF + G2N + G2NF; the abundance of fucosylation (F): G0F + G0NF + G0-NF + G1F + G1FS + G1NF + G1-NF + G2F + G2FS + G2NF; the abundance of fucosylation of neutral glycans (F neutral, FN): G0F + G0NF + G0-NF + G1F + G1NF + G1-NF + G2F + G2NF; the abundance of fucosylation of sialylated glycans (F sialo, FS): G1FS + G2FS; and the abundance of sialylation (S): G1S + G1FS + G2FS + G2S. Relative quantification of IgG galactosylation was measured by a Gal-ratio formula: G0/(G1 + G2 × 2); the abundance of agalactosylation (G0): G0 + G0F + G0N + G0NF + G0-NF; the abundance of monogalactosylation (G1): G1 + G1F + G1FS + G1-N +G1N + G1NF + G1-NF + G1NFS + G1S; and the abundance of digalactosylation (G2): G2 + G2F + G2FS +G2N + G2NF + G2NFS + G2S + G2S2.

### Model Construction

We used F-test to select features that had significant difference between the healthy and migraine groups using Pandas 1.0.5 (27) and scikit-learn 0.23.1 (28) in Python 3.8.3. The top seven features were chosen. To optimize the model parameters efficiently, all the values of the features (N-glycopeptide abundances) were normalized by min-max scaling. Then, we constructed a binary classification model with feedforward neural network using PyTorch 1.6.0 (29) in Python 3.8.3 to classify the migraine and healthy groups. The network consisted of one input layer of 7 features (IgG1 G0-NF, IgG1 G2NF, IgG2 G0N, IgG2 G1N, IgG2 G2N, IgG2 G2NF, and IgG3/4 G0), two hidden layers of 6 and 4 neurons both using Sigmoid as activation function, and finally one output layer of 2 neurons followed by function Softmax, representing two results (having migraine or not). When predicting, the network will calculate from the input abundances of featured N-glycopeptides and give two results representing possibilities of each status. The higher one will be considered as the predicted result. The network can express as follows:


$$Y=Softmax(W_{3}{}^{T}Sigmoid(W_{2}{}^{T}Sigmoid(W_{1}{}^{T}X+B_{1})+B_{2})\;+B_{3})$$

Where, **X** = [*x*_1_, *x*_2_, …, *x*_*n*_] represents the n input features, $W_{1}{}^{T}$[shape: (7,6)], $W_{2}{}^{T}$[shape: (6,4)], and $W_{3}{}^{T}$[shape: (4,2)] are three weight matrices and **B**_**1**_[shape: (6,1)], **B**_**2**_[shape: (4,1)], and **B**_**3**_[shape: (2,1)] are three intercept matrices. **Y** = [*y*_1_, *y*_2_] is the predicted possibilities of each status. The expressions of Sigmoid and Softmax are as follows:


$$Sigmoid(x)=\;\frac{1}{1+e^{-x}}\;$$

$$Softmax(x_{i})=\;\frac{e^{x_{i}}}{\sum_{i=1}^{n}\;e^{x_{i}}}\;$$

5-fold cross-validation was applied to verify the reliability of the model due to the limitation of the amount of data. The model's performance was evaluated by the area under the curve (AUC) values (scikit-learn Python module) (30). The dataset was randomly partitioned into five equal-sized subsets, in which four subsets were used as training sets and the other one subset was used as a test set. The training process was executed five times, with each of the divided training set and test set. The receiver operating characteristic (ROC) curves and the AUC values were given by each fold that can represent the generalization ability of the model. The model is completely random (has no prediction ability at all) at an AUC of 0.5 and the higher the average AUC value is, the higher the performance of the model. The model performs perfectly with no mistake in prediction when the average AUC value equals one. The charts were plotted using Python-Matplotlib (31). Finally, a model of the same features, network form, and hyperparameters were applied to the whole dataset as the training set to give the final prediction model.

## Results

### Baseline Clinical Information of the Patients and Control Groups

Twenty patients with migraine and 20 healthy controls were enrolled. In the migraine group, there were 5 patients with aura and 15 patients without aura. Seven patients were in the ictal phase and 13 patients were in the interictal phase. Nine patients with migraine were comorbid with allergic diseases. It has been reported that gender and age have a greater impact on glycosylation (32, 33). Our data indicated that there were no significant differences between the two groups of subjects in the age, gender, family history of headache, and comorbidity of allergic diseases (Table 1).

### Strategy for Immunoglobulin G N-Glycopeptide Analysis With Matrix-Assisted Laser Desorption/Ionization Time-Of-Flight Mass Spectrometry

**Figure 1:**
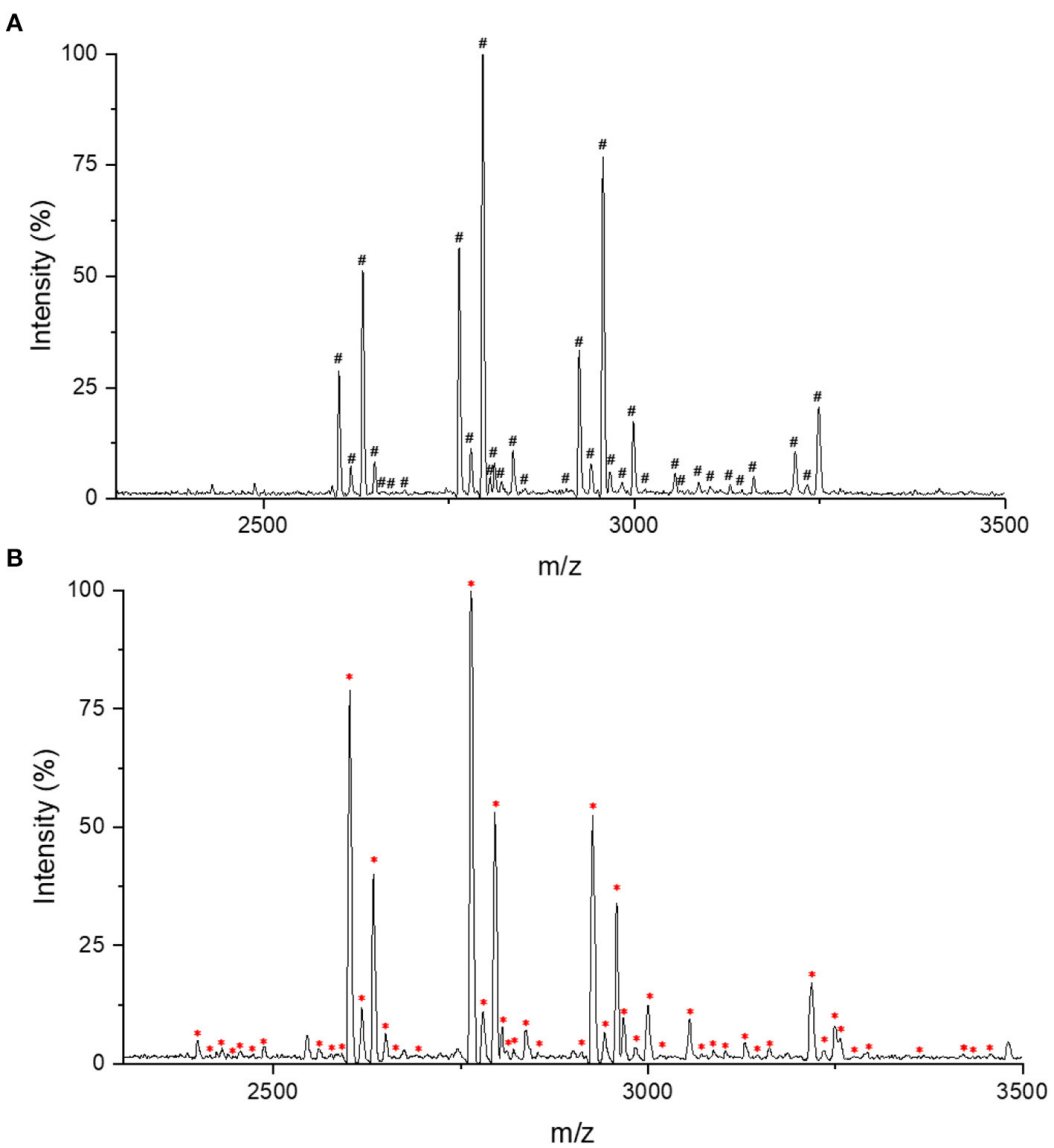
Matrix-assisted laser desorption/ionization time-of-flight mass spectrometry (MALDI-TOF-MS) profiling in the positive-ion linear mode of enriched immunoglobulin G (IgG) N-glycopeptides. **(A)** The IgG N-glycopeptides enriched with chitosan@poly(glycidyl methacrylate)@iminodiacetic acid (CS@PGMA@IDA). **(B)** The IgG N-glycopeptides enriched with poly(glycerol methacrylate)@chitosan (PGMA@CS). ^#^Represents the detected 33 IgG N-glycopeptides by CS@PGMA@IDA. *Represents the detected 49 IgG N-glycopeptides by PGMA@CS.

For IgG intact N-glycopeptides analysis, it is essential to purify N-glycopeptides before MS analysis. On one hand, the presence of nine glycoproteins from the purification process of IgG by protein G will confuse the analyses (25). On the other hand, compared with unmodified peptides, glycopeptides have a lower abundance and relatively lower ionization efficiency, which can also affect the detection (26). In previous reports, we established a high-throughput and effective strategies for obtaining and analyzing IgG intact N-glycopeptides with MALDI-TOF-MS, which used chitosan@poly(glycidyl methacrylate)@iminodiacetic acid (CS@PGMA@IDA) to enrich the N-glycopeptides (25). After comparing the CS@PGMA@IDA with the novel nanosphere PGMA@CS (Figures 1A,B), it can be seen that more IgG N-glycopeptides were detected with PGMA@CS, which shows the high purification and enrichment ability.

**Figure 2:**
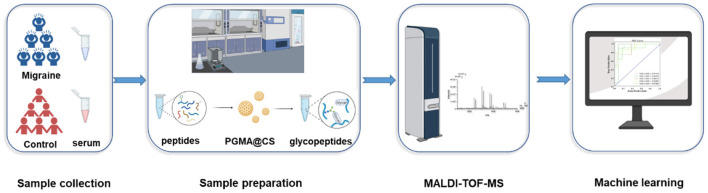
The high-throughput and effective strategies for obtaining and analyzing IgG intact N-glycopeptides for patients with migraine and healthy control subjects.

In this study, we chose to use PGMA@CS instead of CS@PGMA@IDA to improve the IgG N-glycopeptides enrichment ability, optimizing the strategy for migraine cohorts (Figure 2). Overall, after collecting the human serum, the serum preparation was proceeded, including the IgG isolation, tube-gel digestion, and the IgG N-glycopeptides enrichment by PGMA@CS. Then, the purified IgG N-glycopeptides were detected by MALDI-TOF-MS and the data were analyzed by machine learning to explore the potential biomarkers of migraine.

It should be noted that IgG subclasses have a high various abundances and high similar peptide moieties: IgG1, 60%, EEQYNSTYR; IgG2, 32%, EEQFNSTFR; IgG3, 4%, EEQYNSTFR; and IgG4, 4%, EEQFNSTYR (25). All of the N-glycopeptides IgG3 and IgG4 are the isomers, thus the signals of IgG3 and IgG4 N-glycopeptides were considered together. In addition, due to the low abundance of IgG3 and IgG4 and isomers existence, the signal of IgG3/4 N-glycopeptides might overlap with that of other subclasses of IgG N-glycopeptides. Therefore, the IgG3/4 Fc N-glycopeptide was ignored when the signals overlapped (34, 35). A total of 49 N-glycopeptides were detected and considered in this study, as shown in Supplementary Table 1.

### Repeatability

Three standard intravenous immunoglobulins (IVIGs) (5 μg) were used to determine the precision of the analytical workflow. The precision was determined by calculating the relative SDs (RSDs) of intensities of the six minor components (m/z = 2,602.1, 2,634.0, 2,764.1, 2,796.1, 2,926.2, and 2,958.2). It was found that the relative SD was <15.0% over each sample plate (Supplementary Table 2). This error range was in an acceptable range for complex biological sample analysis (36), so no batch correction was performed (25).

### Serum Immunoglobulin G N-Glycopeptides Profiling in Patients With Migraine

**Figure 3:**
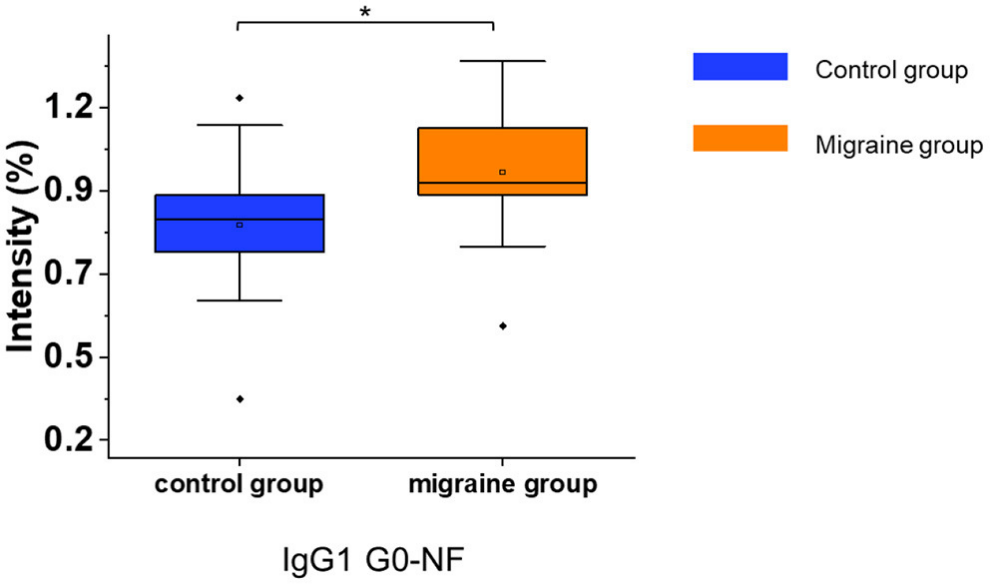
The serum level of IgG N-glycopeptides in the migraine (*n* = 20) and healthy control groups (*n* = 20). The level of IgG N-glycopeptides was presented as box plots showing median values, upper and lower quartiles, as well as the whole range for patients and controls. The glycan species were named as G0, G1, and G2 according to the numbers of galactoses, F, fucose; N, N-acetylglucosamine. *p < 0.05.

After the consistent stability of our strategy was confirmed, the serum IgG N-glycopeptides were analyzed in patients with migraine and healthy controls by this workflow (Supplementary Table 3). The glycopeptide IgG1 G0-NF was increased in patients with migraine compared with the healthy control group (*p* = 0.012, Figure 3) with the two-tailed Student's *t*-test.

**Figure 4:**
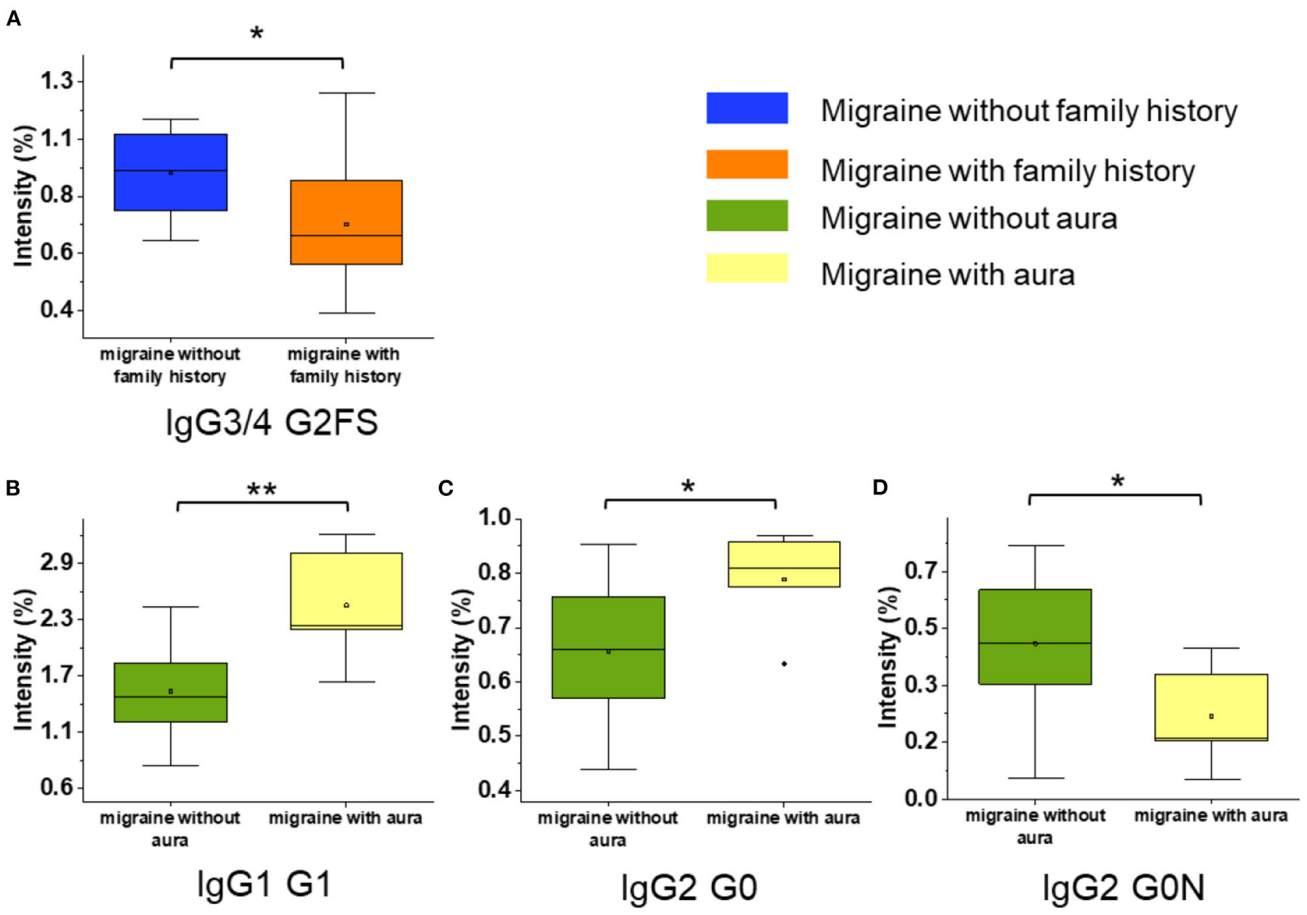
The serum level of IgG glycopeptides in the different migraine subgroups and phases. **(A)** The serum level of IgG3/4 G2FS of migraine with and without family history (*n* = 11/9). **(B–D)** The serum level of IgG glycopeptides of migraine with and without aura (*n* = 5/15). The level of IgG glycopeptides was presented as box plots showing median values, upper and lower quartiles, as well as the whole range for patients and controls. The glycan species were named as G0, G1, and G2 according to the numbers of galactoses, F, fucose; S, sialic acid; N, N-acetylglucosamine. *p < 0.05, **p < 0.01.

Patients with migraine were further grouped based on the phase of migraine, aura, and family history of headache. There was no statistical difference in the gender, age, family history of headache, and comorbidity with allergic diseases between subgroups. Then, we profiled the N-glycopeptides in different subgroups of migraine (Supplementary Table 4). In patients with migraine with family history of headache, the IgG3/4 G2FS (*p* = 0.041, Figure 4A) was significantly decreased. It was found that the serum level of the IgG1 G1 (*p* = 0.004, Figure 4B) and IgG2 G0 (*p* = 0.045, Figure 4C) was increased in patients with migraine with aura, while the serum level of IgG2 G0N (*p* = 0.043, Figure 4D) in patients with migraine with aura was significantly lower than that of patients with migraine without aura. No significant difference in IgG glycopeptides was found between the ictal phase and interictal phase of patients with migraine.

### N-Glycosylation Profiling in Patients With Migraine

The derived N-glycosylation characteristics were also used to distinguish patients with migraine from healthy controls. Considering the isomers exist, the glycosylation of IgG3/4 was not considered to avoid the information mistakes due to neglect. The distribution of the total IgG galactosylation was referred to as Gal-ratio. The higher Gal-ratio reflects the lower content of IgG galactosylation. Other glycan features were annotated in Methods. The summary of changes in the glycan features among the controls, patients with migraine, and patients with migraine subgroup in the discovery set is given in Supplementary Tables 5, 6.

**Figure 5:**
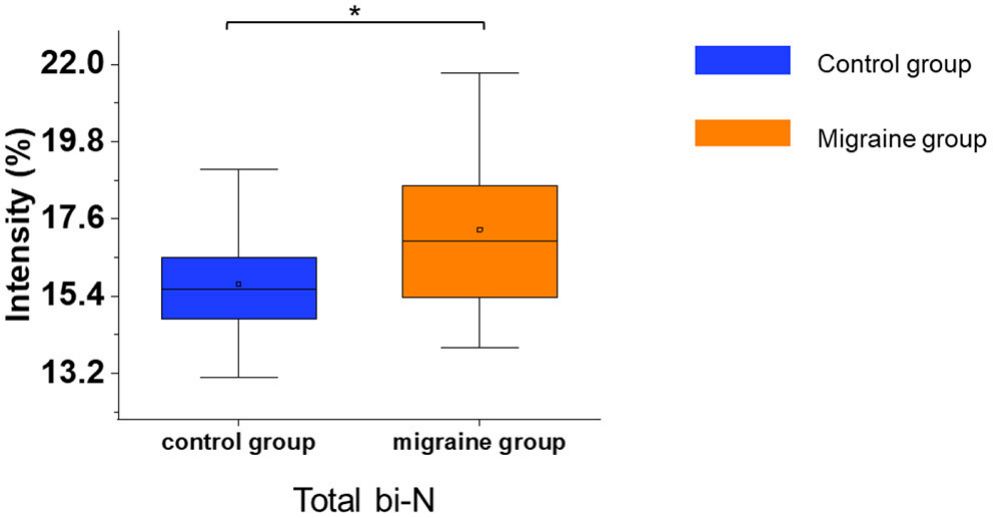
The serum level of IgG N-glycosylation in the migraine (*n* = 20) and control groups (*n* = 20). The serum level of IgG N-glycosylation was presented as box plots showing median values, upper and lower quartiles, as well as the whole range for patients and controls. bi-N, bisecting N-acetylglucosamine. *p < 0.05.

In the serum of patients with migraine, the median of the total bisecting N-acetylglucosamine was significantly increased (*p* = 0.017, Figure 5). Other glycosylations such as fucosylation and sialylation had no significant difference (Supplementary Table 5). In addition, the migraine subgroups were also analyzed for N-glycosylation, but there were no significant differences in N-glycosylation among the migraine subgroups.

### Construction of the Predictive Model to Identify Migraine

**Figure 6:**
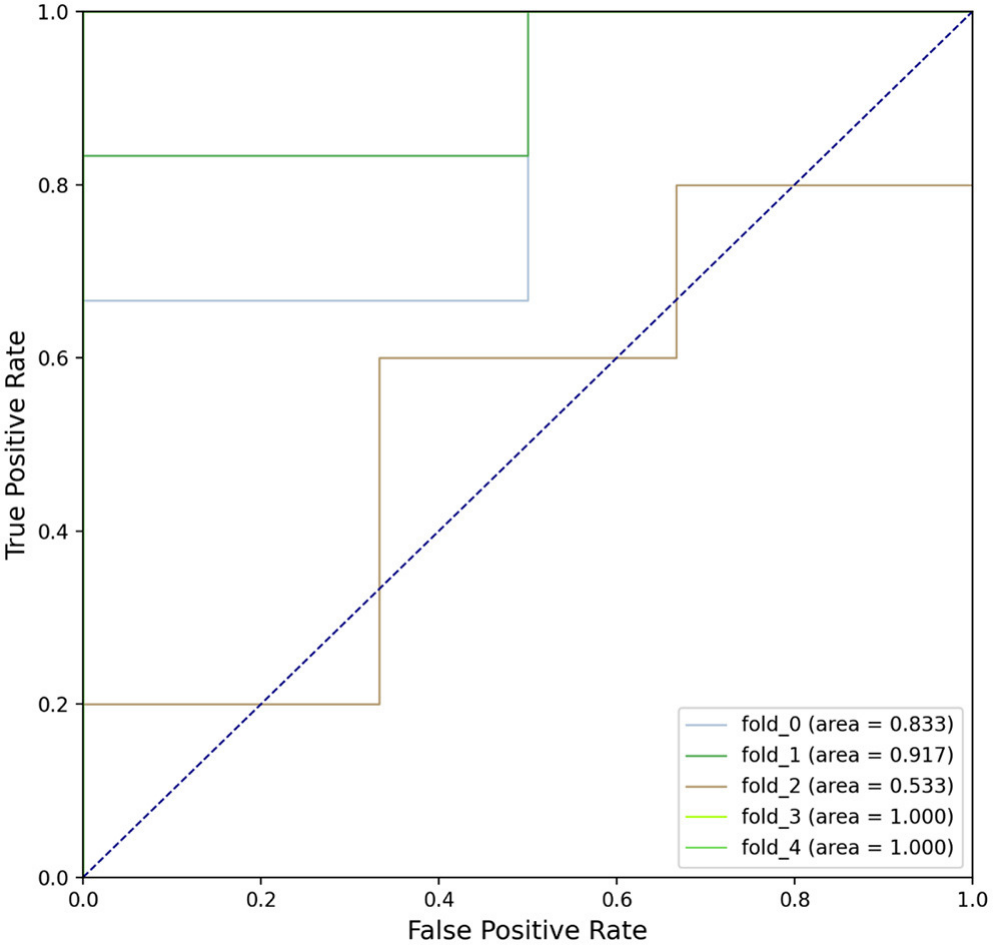
The receiver operating characteristic (ROC) curve of the feedforward neural network (FFNN) model performing in 5-fold cross-validation's test sets; each line represents the prediction of a fold of test samples in the validation.

At present, the diagnosis of migraine still depends on medical history and there is no gold standard for diagnosis. Thus, we constructed a feedforward neural network (FFNN) to distinguish the migraine group from the healthy group. We selected seven N-glycopeptides (IgG1 G0-NF, IgG1 G2NF, IgG2 G0N, IgG2 G1N, IgG2 G2N, IgG2 G2NF, and IgG3/4 G0) as features, constructed a two hidden layer network, and tuned hyperparameters for better performance. Then, 5-fold cross-validation was used to verify the reliability of the model; the ROC curves of validation are shown in Figure 6. The average AUC value of the model was 0.857 (0.833, 0.917, 0.533, 1.000, and 1.000), which indicated that the model had good accuracy in predicting both the migraine and healthy individuals and its performance was stable when training sets and test sets change between different folds. These results indicated that the model had a good performance in identifying migraine individuals. IgG1 G0-NF was overlapped with the differential glycopeptides between the migraine and healthy control groups. The parameters of the model are given in the Supplementary Information “Model Parameters.”

## Discussion

Immunoglobulin G may be involved in the occurrence of migraine, though the specific pathogenesis of migraine is still unclear. IgG N-glycosylation can affect complement-dependent cytotoxicity (CDC), antibody-dependent cellular cytotoxicity (ADCC), antibody-dependent cellular phagocytosis (ADCP), and inflammatory processes (37). Therefore, we focused on the IgG N-glycosylation and N-glycopeptides of migraine with the method of MS, in order to find the biomarkers of migraine, and provide clues for the exploration of the pathophysiological mechanism of migraine.

Mass spectrometry-based approaches are often used for glycoprotein characterization. But, due to the low abundance and relatively low ionization efficiency of glycopeptides compared with unmodified peptides, it is necessary to purify N-glycopeptides before MS analysis. Therefore, we used PGMA@CS to enrich the IgG N-glycopeptides in patients' serum with migraine. The more precise measurements of N-glycopeptides can assist us in better understanding the situation of IgG N-glycosylation in patients with migraine.

Statistical analysis showed that total bisecting N-acetylglucosamine was increased in patients with migraine, while there was no significant change in galactosylation, sialylation, and fucosylation. Bisecting N-acetylglucosamine can enhance antibody FC that binds to the Fcγ receptor IIIa (FcγRIIIa) with higher affinity (38) and enhances the ADCC efficacy of the antibody. Maurice et al. reported that patients with Lambert–Eaton myasthenic syndrome (LEMS) below 50 years showed elevated levels of bisecting N-acetylglucosamine on both the IgG1 and IgG2 (39). LEMS is an immune-related disease with consistent glycosylation changes. We speculated that migraine might also be immune related.

Subgroup analyses were conducted in order to explore the relationship between the migraine aura, phase of migraine, family history, and IgG glycopeptide. In the migraine group, IgG1 G0-NF was higher than that in the healthy control group; IgG1 G1 and IgG2 G0 were higher and IgG2 G0N was lower in patients with migraine with aura than those without aura. IgG3/4 G2FS was lower in patients with migraine with family history of headache than those without family history of headache.

A prediction model of migraine was established. It was found that a group of IgG N-glycopeptides had good prediction performance (IgG1 G0-NF, IgG1 G2NF, IgG2 G0N, IgG2 G1N, IgG2 G2N, IgG2 G2NF, and IgG3/4 G0). The average AUC was 0.857, which indicated that our application of IgG N-glycopeptide model to predict migraine might be reliable. Moreover, we speculated that the serum N-glycopeptide IgG1 G0-NF might be one of the potential biomarkers for the diagnosis of migraine based on our results, but it needs to be tested in a larger sample.

## Conclusion

In summary, the IgG N-glycosylation was analyzed in patients with migraine and healthy controls in this study. The change of serum N-glycosylation profiling in patients with migraine and the different migraine subgroups was explored. Besides, a predictive model of migraine was established, showing a good prediction performance. The relationship between IgG N-glycosylation and migraine has not been reported previously; our data show that it was a possible exploration direction. There is also a major limitation in this study that the sample size was inadequacy and the conclusion needs to be tested in a larger sample. But, this preliminary study suggested that the comprehensive IgG N-glycosylation analysis has the potential to identify useful biomarkers and new therapeutic targets for migraine.

## Data Availability Statement

The original contributions presented in the study are included in the article/Supplementary Materials, further inquiries can be directed to the corresponding authors.

## Ethics Statement

The studies involving human participants were reviewed and approved by Ethical Review Committee of Peking University People's Hospital. The patients/participants provided their written informed consent to participate in this study.

## Author Contributions

HG, YH, JX, and XZ designed the study. YH collected the clinical information and blood samples, and performed the pretreatment of blood samples. XZ and YW performed the MALDI-TOF-MS and data analysis. NL and ZL performed the binary classification model construction. JZ given advises for the study design. JX, HG, YH, YW, NL, and XZ wrote the manuscript. All authors contributed to the article and approved the submitted version.

## Conflict of Interest

The authors declare that the research was conducted in the absence of any commercial or financial relationships that could be construed as a potential conflict of interest.

## Publisher's Note

All claims expressed in this article are solely those of the authors and do not necessarily represent those of their affiliated organizations, or those of the publisher, the editors and the reviewers. Any product that may be evaluated in this article, or claim that may be made by its manufacturer, is not guaranteed or endorsed by the publisher.
