# Supplementary material for: A Preliminary Study on Change of Serum Immunoglobulin G Glycosylation in Patients With Migraine
Source: Front Neurol. 2022 May 23;13:860555. doi: 10.3389/fneur.2022.860555 (PMC9168990; doi:10.3389/fneur.2022.860555)
Supplement: Supplementary file 1 [file Table_1.DOCX]

**Table S1. Theoretical masses of digested Fc N-glycopeptides of human IgG subclasses 1, 2 and 3/4.**

| **Glycan species** | **Glycan Structures** | **IgG1**  P01857*^m^* | **IgG2**  P01859*^m^* | **IgG3/4**  P01860/P01861*^m^* |
| --- | --- | --- | --- | --- |
|  |  | EEQYN^*^STYR | EEQFN^*^STFR | EEQYN^*^STFR/  EEQFN^*^STYR |
|  |  | [M+H]^+^ | [M+H]^+^ | [M+H]^+^ |
| G0 | 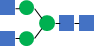 | 2488.0 | 2456.0 | 2472.0 |
| G0F | 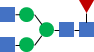 | 2634.0 ^a^ | 2602.1 | 2618.1 ^n^ |
| G0N | 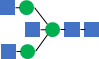 | 2691.1 | 2659.1 | 2675.1  （ND） |
| G0NF | 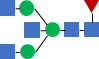 | 2837.1 ^a^ | 2805.1 | 2821.1 ^n^ |
| G0-NF | 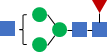 | 2431.0 ^a^ | 2399.0 | 2415.0 ^n^ |
| G1 | 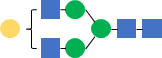 | 2650.0 | 2618.1 ^n^ | 2634.0 ^a^ |
| G1F | 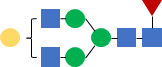 | 2796.1 ^a^ | 2764.1 | 2780.1 ^n^ |
| G1FS | 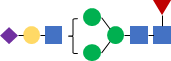 | 3087.2 ^a^ | 3055.2 | 3071.2 ^n^ |
| G1-N | 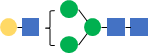 | 2447.0 | 2415.0 ^n^ | 2431.0 ^a^ |
| G1N | 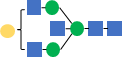 | 2853.1 | 2821.1 ^n^ | 2837.1 ^a^ |
| G1NF | 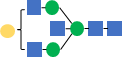 | 2999.2 ^a^ | 2967.2 | 2983.2 ^n^ |
| G1-NF | 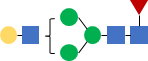 | 2593.0 | 2561.0 | 2577.0 |
| G1NFS | 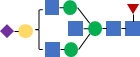 | 3290.3 | 3258.3 | 3274.3 |
| G1S | 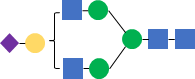 | 2941.1  （ND） | 2909.1 | 2925.1  （ND） |
| G2 | 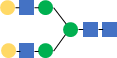 | 2812.1 | 2780.1 ^n^ | 2796.1 ^a^ |
| G2F | 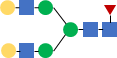 | 2958.2 | 2926.2 | 2942.2 |
| G2FS | 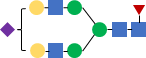 | 3249.2 | 3217.3 | 3233.3 |
| G2N | 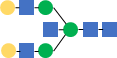 | 3015.2 | 2983.2 ^n^ | 2999.2 ^a^ |
| G2NF | 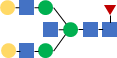 | 3161.2 | 3129.2 | 3145.2 |
| G2NFS | 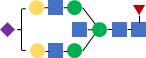 | 3452.3 | 3420.3 | 3436.3 |
| G2S | 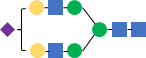 | 3103.2 | 3071.2 ^n^ | 3087.2 ^a^ |
| G2S2 | 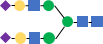 | 3394.3  （ND） | 3362.3 | 3378.3  （ND） |
| \| N-acetylglucosamine Core-fucose Mannose Galactose  Sialylation \| \| --- \| | | | | |

**Note:** *^m^* represents UniProtKB ID. ^*^represents the glycosylation site. *^a^* represents isomeric glycopeptides of IgG1 and IgG3/4. *^n^* represents isomeric glycopeptides of IgG2 and IgG3/4. The glycan species were named as follows: G0, G1, and G2: different numbers of galactoses, F: fucose, N: N-acetylglucosamine, and S: sialic acid. ND: the glycan which was not detected in this study.

**Table S2. RSDs of the average abundance of main glycopeptides in standard IVIGs.**

| Theo. MH+ [Da] | IVIG1 | IVIG2 | IVIG3 | AVERAGE | SD | RSD |
| --- | --- | --- | --- | --- | --- | --- |
| 2602.1 | 11.11% | 11.89% | 11.38% | 11.46% | 0.32% | 2.81% |
| 2634.0 | 8.13% | 7.77% | 6.60% | 7.50% | 0.65% | 8.72% |
| 2764.1 | 15.21% | 16.10% | 15.27% | 15.53% | 0.41% | 2.61% |
| 2796.1 | 11.31% | 11.05% | 8.91% | 10.43% | 1.08% | 10.34% |
| 2926.2 | 8.20% | 8.65% | 8.32% | 8.39% | 0.19% | 2.29% |
| 2958.2 | 6.92% | 6.76% | 5.72% | 6.47% | 0.53% | 8.27% |

**Table S3. Comparison of the** **abundance of IgG Fc N-glycopeptides in** **migraine cohorts compared with those of controls.**

| IgG subclass | Glycans | Control (%)  Average (95%CI) | Migraine（%）  Average (95%CI) | t-value | P-value |
| --- | --- | --- | --- | --- | --- |
| IgG1 | G0 | 1.07 (0.89-1.26) | 1.21 (1.01-1.41) | -1.008 | 0.320 |
|  | G0F | 7.62 (6.54-8.69) | 7.69 (6.25-9.14) | -0.089 | 0.930 |
|  | G0N | 0.8 (0.71-0.89) | 0.85 (0.75-0.95) | -0.675 | 0.504 |
|  | G0NF | 1.6 (1.43-1.76) | 1.75 (1.56-1.94) | -1.278 | 0.209 |
|  | G0-NF | 0.83 (0.75-0.91) | 0.97 (0.89-1.05) | -2.626 | **0.012*** |
|  | G1 | 1.75 (1.52-1.99) | 1.77 (1.49-2.06) | -0.124 | 0.902 |
|  | G1F | 11.73 (10.67-12.78) | 10.9 (9.46-12.34) | 0.944 | 0.351 |
|  | G1FS | 0.67 (0.57-0.77) | 0.68 (0.54-0.82) | -0.129 | 0.898 |
|  | G1-N | 0.39 (0.31-0.47) | 0.38 (0.31-0.45) | 0.264 | 0.793 |
|  | G1N | 0.85 (0.79-0.92) | 0.88 (0.78-0.98) | -0.380 | 0.706 |
|  | G1NF | 2.23 (2.01-2.46) | 2.39 (2.2-2.59) | -1.096 | 0.280 |
|  | G1-NF | 0.44 (0.39-0.5) | 0.47 (0.4-0.54) | -0.584 | 0.563 |
|  | G1NFS | 0.91 (0.81-1.01) | 1.04 (0.9-1.18) | -1.588 | 0.121 |
|  | G2 | 0.86 (0.64-1.08) | 0.83 (0.55-1.11) | 0.178 | 0.860 |
|  | G2F | 7.1 (5.88-8.33) | 6.18 (5.3-7.06) | 1.243 | 0.222 |
|  | G2FS | 2.61 (2.11-3.11) | 2.45 (2.11-2.79) | 0.533 | 0.597 |
|  | G2N | 0.63 (0.55-0.7) | 0.63 (0.56-0.7) | -0.101 | 0.920 |
|  | G2NF | 1.08 (0.97-1.19) | 1.23 (1.1-1.36) | -1.771 | 0.085 |
|  | G2NFS | 0.72 (0.64-0.8) | 0.7 (0.55-0.84) | 0.296 | 0.769 |
|  | G2S | 0.71 (0.59-0.83) | 0.79 (0.64-0.94) | -0.901 | 0.373 |
| IgG2 | G0 | 0.68 (0.57-0.78) | 0.71 (0.64-0.78) | -0.509 | 0.614 |
|  | G0F | 11.18 (9.7-12.65) | 11.24 (9.15-13.32) | -0.046 | 0.963 |
|  | G0N | 0.3 (0.2-0.39) | 0.41 (0.31-0.51) | -1.705 | 0.096 |
|  | G0NF | 0.91 (0.71-1.12) | 1.13 (0.8-1.45) | -1.106 | 0.277 |
|  | G0-NF | 0.98 (0.9-1.05) | 1.02 (0.93-1.11) | -0.773 | 0.444 |
|  | G1 | 1.93 (1.69-2.17) | 2.23 (1.93-2.53) | -1.598 | 0.118 |
|  | G1F | 14.76 (13.29-16.22) | 13.83 (12.33-15.34) | 0.896 | 0.376 |
|  | G1FS | 1.68 (1.55-1.82) | 1.71 (1.54-1.88) | -0.248 | 0.806 |
|  | G1-N | 0.41 (0.34-0.47) | 0.47 (0.4-0.55) | -1.399 | 0.170 |
|  | G1N | 0.72 (0.59-0.84) | 0.88 (0.77-0.99) | -1.972 | 0.056 |
|  | G1NF | 1.29 (1.1-1.48) | 1.35 (1.15-1.54) | -0.418 | 0.678 |
|  | G1-NF | 0.95 (0.88-1.02) | 1 (0.92-1.08) | -0.941 | 0.353 |
|  | G1NFS | 0.07 (0.04-0.11) | 0.07 (0.04-0.11) | 0.083 | 0.935 |
|  | G1S | 0.56 (0.49-0.63) | 0.56 (0.45-0.68) | -0.042 | 0.966 |
|  | G2 | 1.95 (1.67-2.22) | 2.03 (1.71-2.35) | -0.391 | 0.698 |
|  | G2F | 7.01 (6.35-7.66) | 6.97 (5.83-8.11) | 0.055 | 0.957 |
|  | G2FS | 2.42 (2.14-2.71) | 2.65 (2.16-3.15) | -0.820 | 0.418 |
|  | G2N | 0.9 (0.75-1.05) | 1.07 (0.98-1.16) | -1.997 | 0.053 |
|  | G2NF | 1.11 (1.02-1.2) | 1.26 (1.12-1.39) | -1.856 | 0.071 |
|  | G2NFS | 0.36 (0.29-0.43) | 0.29 (0.24-0.34) | 1.633 | 0.111 |
|  | G2S | 0.57 (0.52-0.63) | 0.64 (0.57-0.71) | -1.410 | 0.167 |
|  | G2S2 | 0.3 (0.23-0.37) | 0.36 (0.26-0.47) | -1.087 | 0.284 |
| IgG3/4 | G0 | 0.56 (0.47-0.64) | 0.47 (0.4-0.53) | 1.668 | 0.104 |
|  | G1-NF | 0.38 (0.3-0.46) | 0.39 (0.32-0.45) | -0.200 | 0.842 |
|  | G1NFS | 0.41 (0.35-0.48) | 0.5 (0.4-0.59) | -1.504 | 0.141 |
|  | G2F | 1.34 (1.16-1.51) | 1.27 (1.1-1.45) | 0.545 | 0.589 |
|  | G2FS | 0.84 (0.72-0.96) | 0.82 (0.73-0.92) | 0.297 | 0.768 |
|  | G2NF | 0.6 (0.5-0.7) | 0.66 (0.58-0.75) | -1.018 | 0.315 |
|  | G2NFS | 0.25 (0.19-0.32) | 0.22 (0.17-0.27) | 0.807 | 0.425 |

**Note:** the ratios of significant differences were emphasized with ^*^(^*^: *p*<0.05). The glycan species were named as follows: G0, G1, and G2: different numbers of galactoses, F: fucose, N: N-acetylglucosamine, and S: sialic acid.

**Table S4. Description of individual IgG glycopeptides among the subgroups of migraine.**

| Glycan species | Migraine in the interictal phase (%)  Average (95%CI) | Migraine in the ictal phase (%)  Average (95%CI) | t value | P value | Migraine without aura (%)  Average (95%CI) | Migraine with aura (%)  Average (95%CI) | t value | P value | Migraine without family history (%)  Average (95%CI) | Migraine with family history (%)  Average (95%CI) | T value | P value |
| --- | --- | --- | --- | --- | --- | --- | --- | --- | --- | --- | --- | --- |
| **IgG1** |  |  |  |  |  |  |  |  |  |  |  |  |
| G0 | 1.28 (0.99-1.57) | 1.08 (0.84-1.32) | 0.947 | 0.356 | 1.08 (0.94-1.22) | 1.60 (0.88- 2.31) | -1.753 | 0.147 | 1.16 (0.97-1.36) | 1.24 (0.89-1.59) | -0.392 | 0.700 |
| G0F | 8.33 (6.14-10.53) | 6.5 (5.88-7.12) | 1.689 | 0.114 | 7.65 (5.85- 9.45) | 7.82 (4.73- 10.92) | -0.102 | 0.920 | 7.88 (6.34-9.42) | 7.54 (5.03-10.05) | 0.233 | 0.818 |
| G0N | 0.86 (0.73-1) | 0.82 (0.65-0.99) | 0.399 | 0.694 | 0.80 (0.69- 0.91) | 0.99 (0.76- 1.21) | -1.719 | 0.103 | 0.89 (0.71-1.08) | 0.81 (0.69-0.93) | 0.846 | 0.408 |
| G0NF | 1.84 (1.6-2.09) | 1.58 (1.29-1.86) | 1.440 | 0.167 | 1.72 (1.50- 1.95) | 1.83 (1.41- 2.26) | -0.507 | 0.618 | 1.91 (1.64-2.17) | 1.62 (1.36-1.88) | 1.635 | 0.119 |
| G0-NF | 0.96 (0.89-1.04) | 0.99 (0.77-1.2) | -0.220 | 0.831 | 0.96 (0.85- 1.06) | 1.02 (0.93- 1.11) | -0.665 | 0.514 | 0.94 (0.8-1.09) | 1 (0.89-1.1) | -0.668 | 0.512 |
| G1 | 1.8 (1.39-2.22) | 1.72 (1.31-2.14) | 0.251 | 0.805 | 1.56 (1.30- 1.82) | 2.43 (1.75- 3.11) | -3.268 | 0.004 | 1.68 (1.32-2.04) | 1.85 (1.38-2.33) | -0.593 | 0.560 |
| G1F | 11.13 (9.1-13.16) | 10.48 (8.24-12.71) | 0.429 | 0.673 | 10.35 (8.71- 12.00) | 12.55 (9.09- 16.00) | -1.380 | 0.185 | 10.54 (9.11-11.97) | 11.19 (8.66-13.72) | -0.450 | 0.658 |
| G1FS | 0.68 (0.48-0.89) | 0.67 (0.5-0.85) | 0.070 | 0.945 | 0.67 (0.48- 0.86) | 0.71 (0.59- 0.82) | -0.215 | 0.832 | 0.63 (0.37-0.89) | 0.72 (0.55-0.89) | -0.605 | 0.553 |
| G1-N | 0.37 (0.29-0.46) | 0.39 (0.24-0.54) | -0.241 | 0.812 | 0.38 (0.29- 0.47) | 0.37 (0.21- 0.53) | 0.084 | 0.934 | 0.36 (0.29-0.42) | 0.39 (0.27-0.52) | -0.558 | 0.586 |
| G1N | 0.84 (0.72-0.97) | 0.94 (0.75-1.13) | -0.908 | 0.376 | 0.84 (0.72- 0.97) | 0.97 (0.79- 1.16) | -1.181 | 0.253 | 0.83 (0.65-1.02) | 0.91 (0.79-1.03) | -0.819 | 0.423 |
| G1NF | 2.41 (2.22-2.6) | 2.37 (1.85-2.9) | 0.177 | 0.862 | 2.30 (2.09- 2.51) | 2.67 (2.18- 3.17) | -1.769 | 0.094 | 2.46 (2.15-2.78) | 2.34 (2.06-2.62) | 0.637 | 0.532 |
| G1-NF | 0.47 (0.4-0.53) | 0.47 (0.29-0.64) | -0.019 | 0.985 | 0.47 (0.39- 0.55) | 0.46 (0.32- 0.61) | 0.095 | 0.925 | 0.46 (0.4-0.52) | 0.47 (0.35-0.6) | -0.212 | 0.835 |
| G1NFS | 0.99 (0.83-1.14) | 1.14 (0.83-1.45) | -1.119 | 0.278 | 1.04 (0.88- 1.20) | 1.03 (0.65- 1.41) | 0.064 | 0.950 | 1 (0.82-1.19) | 1.07 (0.85-1.29) | -0.496 | 0.626 |
| G2 | 0.83 (0.42-1.23) | 0.83 (0.41-1.24) | -0.002 | 0.998 | 0.70 (0.39- 1.01) | 1.21 (0.51- 1.91) | -1.670 | 0.112 | 0.63 (0.34-0.92) | 0.99 (0.52-1.46) | -1.329 | 0.200 |
| G2F | 6.08 (4.93-7.22) | 6.38 (4.68-8.09) | -0.333 | 0.743 | 5.92 (5.04- 6.80) | 6.98 (4.02- 9.94) | -1.070 | 0.299 | 5.92 (4.77-7.06) | 6.4 (4.98-7.83) | -0.552 | 0.588 |
| G2FS | 2.36 (1.94-2.78) | 2.63 (1.93-3.32) | -0.768 | 0.452 | 2.38 (1.98- 2.79) | 2.66 (1.83- 3.49) | -0.715 | 0.484 | 2.33 (1.9-2.75) | 2.55 (1.99-3.11) | -0.647 | 0.526 |
| G2N | 0.6 (0.53-0.67) | 0.69 (0.52-0.85) | -1.216 | 0.240 | 0.62 (0.53- 0.71) | 0.67 (0.56- 0.77) | -0.581 | 0.568 | 0.61 (0.51-0.71) | 0.65 (0.54-0.75) | -0.486 | 0.633 |
| G2NF | 1.19 (1.06-1.32) | 1.29 (0.96-1.63) | -0.759 | 0.458 | 1.21 (1.04- 1.39) | 1.27 (1.15- 1.38) | -0.376 | 0.711 | 1.18 (1.01-1.36) | 1.26 (1.06-1.47) | -0.618 | 0.544 |
| G2NFS | 0.65 (0.51-0.8) | 0.77 (0.4-1.14) | -0.790 | 0.440 | 0.68 (0.50- 0.86) | 0.75 (0.45- 1.06) | -0.434 | 0.670 | 0.6 (0.42-0.77) | 0.78 (0.55-1.01) | -1.291 | 0.213 |
| G2S | 0.82 (0.67-0.98) | 0.73 (0.38-1.08) | 0.636 | 0.533 | 0.73 (0.55- 0.91) | 0.96 (0.74- 1.18) | -1.421 | 0.172 | 0.74 (0.53-0.95) | 0.83 (0.61-1.06) | -0.644 | 0.528 |
| **IgG2** | | | |  |  |  |  |  |  |  |  |  |
| G0 | 0.68 (0.58-0.78) | 0.75 (0.66-0.85) | -0.952 | 0.354 | 0.67 (0.59- 0.75) | 0.83 (0.70- 0.95) | -2.156 | 0.045 | 0.7 (0.61-0.8) | 0.71 (0.59-0.83) | -0.124 | 0.903 |
| G0F | 11.14 (8.44-13.84) | 11.42 (7.31-15.53) | -0.126 | 0.901 | 11.64 (9.01- 14.28) | 10.01 (6.13- 13.89) | 0.681 | 0.504 | 11.13 (9.42-12.84) | 11.32 (7.52-15.13) | -0.100 | 0.921 |
| G0N | 0.4 (0.28-0.52) | 0.43 (0.24-0.63) | -0.343 | 0.735 | 0.46 (0.36- 0.57) | 0.25 (0.07- 0.42) | 2.174 | 0.043 | 0.47 (0.4-0.55) | 0.36 (0.19-0.53) | 1.278 | 0.223 |
| G0NF | 1.16 (0.71-1.61) | 1.06 (0.52-1.6) | 0.293 | 0.773 | 1.22 (0.87- 1.57) | 0.85 (-0.16- 1.85) | 1.000 | 0.330 | 1.34 (0.91-1.77) | 0.95 (0.44-1.46) | 1.229 | 0.235 |
| G0-NF | 0.99 (0.87-1.12) | 1.07 (0.94-1.21) | -0.852 | 0.405 | 1.03 (0.93- 1.13) | 1.00 (0.71- 1.29) | 0.317 | 0.755 | 1.02 (0.94-1.1) | 1.03 (0.86-1.19) | -0.128 | 0.900 |
| G1 | 2.35 (1.92-2.79) | 2 (1.68-2.33) | 1.136 | 0.271 | 2.28 (1.89- 2.67) | 2.07 (1.61- 2.54) | 0.600 | 0.556 | 2.49 (2.03-2.95) | 2.02 (1.62-2.42) | 1.660 | 0.114 |
| G1F | 13.49 (11.57-15.42) | 14.47 (11.53-17.4) | -0.618 | 0.544 | 14.32 (12.35- 16.28) | 12.39 (11.16- 13.61) | 1.141 | 0.269 | 13.83 (12.59-15.07) | 13.84 (11.09-16.58) | -0.004 | 0.997 |
| G1FS | 1.67 (1.42-1.92) | 1.77 (1.55-2) | -0.632 | 0.535 | 1.73 (1.54- 1.92) | 1.65 (1.13- 2.18) | 0.364 | 0.720 | 1.71 (1.53-1.89) | 1.71 (1.41-2.01) | 0.022 | 0.983 |
| G1-N | 0.47 (0.36-0.58) | 0.48 (0.38-0.59) | -0.136 | 0.893 | 0.49 (0.41- 0.58) | 0.41 (0.20- 0.62) | 0.944 | 0.358 | 0.47 (0.39-0.56) | 0.47 (0.34-0.61) | -0.009 | 0.993 |
| G1N | 0.9 (0.73-1.06) | 0.84 (0.71-0.98) | 0.439 | 0.666 | 0.91 (0.79- 1.04) | 0.78 (0.46- 1.10) | 1.051 | 0.307 | 0.98 (0.87-1.09) | 0.79 (0.61-0.98) | 1.782 | 0.092 |
| G1NF | 1.35 (1.06-1.63) | 1.35 (1.09-1.61) | -0.012 | 0.991 | 1.41 (1.19- 1.63) | 1.15 (0.68- 1.62) | 1.207 | 0.243 | 1.47 (1.21-1.72) | 1.25 (0.95-1.54) | 1.179 | 0.254 |
| G1-NF | 0.97 (0.85-1.08) | 1.06 (0.96-1.16) | -1.088 | 0.291 | 1.01 (0.91- 1.11) | 0.96 (0.80- 1.13) | 0.532 | 0.601 | 1.03 (0.93-1.13) | 0.98 (0.84-1.11) | 0.604 | 0.553 |
| G1NFS | 0.08 (0.03-0.14) | 0.05 (0-0.1) | 0.974 | 0.343 | 0.06 (0.03- 0.09) | 0.11 (-0.02- 0.25) | -0.932 | 0.397 | 0.07 (0.02-0.13) | 0.07 (0.02-0.13) | -0.015 | 0.988 |
| G1S | 0.55 (0.41-0.69) | 0.58 (0.33-0.91) | -0.272 | 0.789 | 0.55 (0.41- 0.68) | 0.60 (0.3- 0.90) | -0.380 | 0.708 | 0.6 (0.48-1.08) | 0.53 (0.34-0.87) | 0.560 | 0.582 |
| G2 | 2.08 (1.62-2.55) | 1.93 (1.46-3.39) | 0.455 | 0.654 | 2.08 (1.66- 2.49) | 1.89 (1.38- 2.39) | 0.503 | 0.621 | 2.23 (1.97-4.2) | 1.86 (1.3-3.16) | 1.160 | 0.261 |
| G2F | 6.75 (5.06-8.44) | 7.38 (6-13.38) | -0.528 | 0.604 | 7.30 (5.91- 8.70) | 5.97 (3.68- 8.26) | 1.038 | 0.313 | 6.78 (5.56-12.34) | 7.13 (5.15-12.27) | -0.301 | 0.767 |
| G2FS | 2.57 (1.9-3.25) | 2.8 (1.97-4.77) | -0.433 | 0.670 | 2.77 (2.16- 3.37) | 2.31 (1.28- 3.34) | 0.811 | 0.428 | 2.52 (2.02-4.54) | 2.76 (1.9-4.66) | -0.532 | 0.603 |
| G2N | 1.04 (0.92-1.17) | 1.12 (0.96-2.08) | -0.787 | 0.442 | 1.06 (0.94- 1.18) | 1.10 (0.93- 1.26) | -0.330 | 0.745 | 1.14 (1.02-2.16) | 1.01 (0.87-1.88) | 1.558 | 0.137 |
| G2NF | 1.22 (1.05-1.39) | 1.33 (1.07-2.41) | -0.842 | 0.411 | 1.26 (1.10- 1.43) | 1.23 (0.93- 1.54) | 0.189 | 0.852 | 1.24 (1.07-2.31) | 1.27 (1.05-2.32) | -0.217 | 0.831 |
| G2NFS | 0.27 (0.2-0.33) | 0.34 (0.25-0.6) | -1.521 | 0.146 | 0.31 (0.24- 0.37) | 0.25 (0.18- 0.33) | 0.898 | 0.381 | 0.31 (0.24-0.55) | 0.28 (0.2-0.48) | 0.703 | 0.491 |
| G2S | 0.63 (0.54-0.73) | 0.65 (0.53-1.18) | -0.159 | 0.876 | 0.63 (0.53- 0.72) | 0.68 (0.57- 0.79) | -0.647 | 0.526 | 0.63 (0.52-1.15) | 0.65 (0.54-1.19) | -0.295 | 0.771 |
| G2S2 | 0.33 (0.24-0.43) | 0.42 (0.15-0.58) | -0.879 | 0.391 | 0.34 (0.22- 0.47) | 0.43 (0.22- 0.63) | -0.708 | 0.488 | 0.35 (0.22-0.57) | 0.38 (0.21-0.59) | -0.264 | 0.795 |
| **IgG3/4** | | | |  |  |  |  |  |  |  |  |  |
| G0 | 0.49 (0.4-0.58) | 0.43 (0.32-0.76) | 0.744 | 0.467 | 0.48 (0.40- 0.56) | 0.44 (0.28- 0.60) | 0.445 | 0.661 | 0.49 (0.4-0.89) | 0.45 (0.34-0.8) | 0.483 | 0.635 |
| G1-NF | 0.4 (0.32-0.48) | 0.37 (0.24-0.61) | 0.374 | 0.713 | 0.42 (0.34- 0.49) | 0.31 (0.15- 0.47) | 1.556 | 0.137 | 0.42 (0.33-0.75) | 0.36 (0.26-0.63) | 0.822 | 0.422 |
| G1NFS | 0.51 (0.39-0.63) | 0.46 (0.28-0.74) | 0.477 | 0.639 | 0.50 (0.39- 0.61) | 0.49 (0.21- 0.76) | 0.112 | 0.912 | 0.54 (0.43-0.97) | 0.46 (0.3-0.76) | 0.770 | 0.451 |
| G2F | 1.29 (1.04-1.55) | 1.24 (1.02-2.26) | 0.279 | 0.783 | 1.30 (1.07- 1.53) | 1.18 (0.98- 1.39) | 0.595 | 0.559 | 1.41 (1.21-2.62) | 1.16 (0.89-2.05) | 1.562 | 0.136 |
| G2FS | 0.82 (0.67-0.96) | 0.83 (0.71-1.54) | -0.156 | 0.878 | 0.82 (0.70- 0.95) | 0.82 (0.67- 0.97) | 0.053 | 0.958 | 0.93 (0.81-1.74) | 0.74 (0.6-1.33) | 2.198 | 0.041 |
| G2NF | 0.66 (0.55-0.78) | 0.66 (0.52-1.18) | 0.046 | 0.964 | 0.67 (0.57- 0.76) | 0.66 (0.39- 0.92) | 0.100 | 0.921 | 0.69 (0.6-1.29) | 0.64 (0.5-1.14) | 0.592 | 0.561 |
| G2NFS | 0.23 (0.16-0.29) | 0.21 (0.09-0.29) | 0.403 | 0.692 | 0.21 (0.15- 0.28) | 0.24 (0.11- 0.38) | -0.509 | 0.617 | 0.26 (0.17-0.43) | 0.19 (0.12-0.31) | 1.336 | 0.198 |

Note: P values result from Student *t* test. G0, G1 and G2: different numbers of galactoses, F: fucose, N: N-acetylglucosamine and S: sialic acid. Glycan structures are annotated as described in **Table S1**

**Table S5. Comparison of the abundance of IgG N-glycosylation features in migraine patients and controls.**

| Glycosylation features | IgG subclass | Control (%)  Average (95%CI) | Migraine（%）  Average (95%CI) | *t*-value | *P*-value |
| --- | --- | --- | --- | --- | --- |
| Bi-N | IgG1 | 8.82 (8.27-9.38) | 9.46 (8.73-10.19) | -1.430 | 0.161 |
|  | IgG2 | 5.66 (5.01-6.31) | 6.45 (5.87-7.03) | -1.863 | 0.070 |
|  | Total | 15.74 (15.07-16.42) | 17.29 (16.22-18.37) | -2.493 | **0.018*** |
| F | IgG1 | 37.53 (34.28-40.78) | 36.46 (33.24-39.67) | 0.480 | 0.634 |
|  | IgG2 | 42.72 (39.04-46.4) | 42.52 (38.4-46.64) | 0.076 | 0.940 |
|  | Total | 84.08 (82.95-85.21) | 82.84 (81.8-83.87) | 1.655 | 0.106 |
| F neural  (FN) | IgG1 | 32.62 (29.76-35.49) | 31.59 (28.43-34.75) | 0.494 | 0.624 |
|  | IgG2 | 38.18 (34.74-41.62) | 37.79 (33.86-41.72) | 0.152 | 0.880 |
|  | Total | 73.12 (71.62-74.61) | 71.7 (69.9-73.5) | 1.233 | 0.225 |
| F sialo  (FS) | IgG1 | 4.91 (4.27-5.55) | 4.87 (4.31-5.43) | 0.097 | 0.923 |
|  | IgG2 | 4.54 (4.12-4.97) | 4.73 (4.07-5.39) | -0.482 | 0.633 |
|  | Total | 10.96 (10.16-11.76) | 11.13 (9.96-12.31) | -0.248 | 0.805 |
| Gal | IgG1 | 0.27 (0.23-0.31) | 0.3 (0.25-0.34) | -0.900 | 0.374 |
|  | IgG2 | 0.27 (0.24-0.3) | 0.29 (0.23-0.35) | -0.431 | 0.670 |
|  | Total | 0.26 (0.23-0.29) | 0.28 (0.23-0.32) | -0.687 | 0.497 |
| G0 | IgG1 | 11.91 (10.64-13.19) | 12.47 (10.83-14.12) | -0.548 | 0.587 |
|  | IgG2 | 14.04 (12.31-15.77) | 14.5 (12.07-16.93) | -0.314 | 0.755 |
|  | Total | 26.51 (24.37-28.65) | 27.44 (24.53-30.35) | -0.525 | 0.603 |
| G1’ | IgG1 | 18.98 (17.64-20.32) | 18.51 (16.81-20.22) | 0.439 | 0.663 |
|  | IgG2 | 22.36 (20.62-24.11) | 22.1 (20.46-23.75) | 0.220 | 0.827 |
|  | Total | 42.13 (41.29-42.97) | 41.5 (40.86-42.14) | 1.220 | 0.230 |
| G2’ | IgG1 | 13.71 (11.73-15.68) | 12.81 (11.09-14.52) | 0.701 | 0.488 |
|  | IgG2 | 14.62 (13.65-15.59) | 15.27 (13.6-16.95) | -0.692 | 0.494 |
|  | Total | 31.36 (29.07-33.64) | 31.06 (27.99-34.13) | 0.160 | 0.874 |
| S | IgG1 | 5.62 (4.94-6.29) | 5.66 (4.98-6.33) | -0.091 | 0.928 |
|  | IgG2 | 6.27 (5.77-6.78) | 6.66 (5.77-7.54) | -0.768 | 0.447 |
|  | Total | 13.4 (12.49-14.31) | 13.85 (12.32-15.39) | -0.520 | 0.607 |

**Note:** the ratios of significant differences were emphasized with ^*^(^*^: *p*<0.05, ^**^: *p*<0.01). bi-N: bisecting N-acetylglucosamine; F: fucosylation; F neural: fucosylation of neutral glycans, F sialo: fucosylation of sialylated glycans, G： galactosylation, G0： agalactosylation, G1’: monogalactosylation; G2’: digalactosylation; S: sialylation.

**Table S6. Description of individual IgG glycosylation among the subgroups of migraine.**

| Glyco-sylation | Migraine in the interictal phase (%)  Average (95%CI) | Migraine in the ictal phase (%)  Average (95%CI) | t value | P value | Migraine without aura (%)  Average (95%CI) | | Migraine with aura (%)  Average (95%CI) | t value | P value | Migraine without family history (%)  Average (95%CI) | Migraine with family history (%)  Average (95%CI) | t value | P value |
| --- | --- | --- | --- | --- | --- | --- | --- | --- | --- | --- | --- | --- | --- |
| **IgG1** | | | | | | | | | | | | | |
| G0 | 13.28 (10.82-15.74) | 10.97 (10.02-11.92) | 1.853 | 0.084 | 12.21 (10.23- 14.19) | 13.25 (9.27- 17.23) | | -0.549 | 0.590 | 12.79 (11.27-14.31) | 12.21 (9.28-15.15) | 0.348 | 0.732 |
| G1 | 18.69 (16.4-20.98) | 18.19 (15.08-21.29) | 0.280 | 0.783 | 17.91 (15.75- 20.08) | 19.63 (16.38- 22.87) | | -1.992 | 0.062 | 17.97 (16.06-19.89) | 18.96 (16.05-21.86) | -0.574 | 0.573 |
| G2 | 12.53 (10.4-14.66) | 13.32 (9.74-16.9) | -0.439 | 0.666 | 11.75 (9.62- 13.88) | 14.76 (11.91- 17.61) | | -1.171 | 0.257 | 12 (9.87-14.14) | 13.46 (10.68-16.25) | -0.856 | 0.403 |
| Gal | 0.32 (0.25-0.38) | 0.26 (0.2-0.31) | 1.276 | 0.218 | 0.32 (0.26- 0.38) | 0.25 (0.17- 0.33) | | 0.482 | 0.636 | 0.32 (0.25-0.39) | 0.28 (0.21-0.35) | 0.857 | 0.403 |
| F | 37.09 (32.7-41.48) | 35.28 (29.84-40.72) | 0.538 | 0.597 | 35.82 (31.52- 40.11) | 37.65 (31.91- 43.40) | | -1.225 | 0.236 | 35.86 (32.95-38.76) | 36.95 (31.19-42.71) | -0.336 | 0.740 |
| FN | 32.41 (27.98-36.85) | 30.06 (25.25-34.88) | 0.712 | 0.485 | 31.35 (27.23- 35.47) | 32.04 (25.89- 38.18) | | -1.131 | 0.273 | 31.3 (28.56-34.03) | 31.83 (26.1-37.56) | -0.167 | 0.869 |
| FS | 4.68 (4.02-5.34) | 5.22 (4.01-6.42) | -0.935 | 0.362 | 4.47 (3.83- 5.10) | 5.61 (4.62- 6.61) | | -0.587 | 0.565 | 4.56 (3.84-5.29) | 5.12 (4.24-6) | -1.013 | 0.325 |
| Bi-N | 9.39 (8.67-10.11) | 9.6 (7.69-17.29) | -0.274 | 0.787 | 8.95 (8.04- 9.87) | 10.41 (9.33- 19.75) | | -1.176 | 0.255 | 9.49 (8.26-17.76) | 9.44 (8.42-17.86) | 0.067 | 0.947 |
| S | 5.5 (4.73-6.28) | 5.95 (4.42-7.47) | -0.627 | 0.539 | 5.15 (4.37- 5.92) | 6.61 (5.49- 7.73) | | -0.787 | 0.442 | 5.3 (4.44-6.17) | 5.95 (4.88-7.02) | -0.979 | 0.340 |
| **IgG2** | | | | | | | | | | | | | |
| G0 | 14.37 (11.17-17.58) | 14.74 (10.12-19.35) | -0.141 | 0.890 | 15.60 (12.22- 18.99) | 12.45 (9.18- 15.72) | | 0.752 | 0.462 | 14.66 (12.64-16.68) | 14.37 (9.95-18.8) | 0.125 | 0.902 |
| G1 | 21.83 (19.71-23.96) | 22.61 (19.4-25.82) | -0.447 | 0.660 | 22.61 (20.34- 24.89) | 21.16 (18.59- 23.74) | | 1.451 | 0.164 | 22.65 (21.03-24.26) | 21.66 (18.77-24.55) | 0.597 | 0.558 |
| G2 | 14.9 (12.58-17.22) | 15.97 (13.29-18.65) | -0.614 | 0.547 | 14.95 (12.97- 16.94) | 15.86 (12.18- 19.55) | | 0.998 | 0.331 | 15.2 (13.26-17.14) | 15.33 (12.48-18.18) | -0.078 | 0.938 |
| Gal | 0.29 (0.21-0.38) | 0.27 (0.19-0.36) | 0.309 | 0.761 | 0.31 (0.22- 0.39) | 0.25 (0.15- 0.35) | | 0.077 | 0.939 | 0.28 (0.23-0.34) | 0.29 (0.18-0.4) | -0.137 | 0.893 |
| F | 41.67 (36.06-47.27) | 44.1 (37.05-51.14) | -0.564 | 0.580 | 43.49 (37.68- 49.29) | 40.72 (34.51- 46.92) | | 1.351 | 0.193 | 42.44 (39.44-45.44) | 42.58 (34.96-50.19) | -0.033 | 0.974 |
| FN | 37.06 (31.83-42.3) | 39.13 (31.99-46.28) | -0.502 | 0.622 | 39.03 (33.33- 44.72) | 35.49 (30.52- 40.46) | | 1.289 | 0.214 | 37.83 (35-40.65) | 37.76 (30.48-45.04) | 0.018 | 0.986 |
| FS | 4.6 (3.69-5.51) | 4.96 (3.88-6.04) | -0.526 | 0.605 | 4.46 (3.81- 5.11) | 5.23 (3.59- 6.86) | | 0.699 | 0.493 | 4.61 (3.97-5.26) | 4.82 (3.66-5.98) | -0.335 | 0.742 |
| Bi-N | 6.41 (5.62-7.2) | 6.53 (5.52-12.05) | -0.193 | 0.849 | 6.62 (6.00- 7.23) | 6.14 (4.75- 10.89) | | 1.544 | 0.140 | 7.03 (6.2-13.23) | 5.98 (5.21-11.19) | 1.993 | 0.062 |
| S | 6.45 (5.32-7.57) | 7.04 (5.3-8.79) | -0.641 | 0.529 | 6.16 (5.35- 6.97) | 7.58 (5.39- 9.77) | | 0.252 | 0.804 | 6.54 (5.67-7.4) | 6.76 (5.19-8.32) | -0.263 | 0.796 |
| **Total** | | | | | | | | | | | | | |
| G0 | 28.14 (24.04-32.24) | 26.14 (21.72-30.56) | 0.658 | 0.519 | 28.80 (25.24- 32.35) | 24.92 (19.32- 30.52) | | 0.322 | 0.751 | 27.93 (24.62-31.24) | 27.04 (22.06-32.02) | 0.320 | 0.753 |
| G1 | 41.43 (40.62-42.25) | 41.63 (40.34-42.92) | -0.294 | 0.772 | 41.39 (40.60- 42.18) | 41.71 (40.38- 43.04) | | -1.153 | 0.264 | 41.57 (40.6-42.55) | 41.44 (40.48-42.41) | 0.202 | 0.842 |
| G2 | 30.43 (26.26-34.6) | 32.23 (26.98-37.48) | -0.561 | 0.581 | 29.82 (25.90- 33.73) | 33.37 (27.78- 38.96) | | -0.073 | 0.943 | 30.49 (27.08-33.91) | 31.52 (26.25-36.79) | -0.349 | 0.731 |
| Gal | 0.29 (0.22-0.36) | 0.25 (0.18-0.32) | 0.733 | 0.473 | 0.30 (0.24- 0.36) | 0.24 (0.16- 0.32) | | 0.284 | 0.780 | 0.28 (0.22-0.33) | 0.28 (0.2-0.36) | 0.050 | 0.961 |
| F | 82.67 (81.26-84.08) | 83.15 (81.39-84.92) | -0.450 | 0.658 | 83.27 (81.83- 84.71) | 82.03 (80.60- 83.46) | | 1.806 | 0.088 | 82.54 (81.44-83.65) | 83.08 (81.29-84.86) | -0.515 | 0.613 |
| FN | 71.83 (69.45-74.2) | 71.47 (68.07-139.54) | 0.188 | 0.853 | 72.83 (70.47- 75.18) | 69.62 (67.10- 136.72) | | 0.908 | 0.376 | 71.65 (69.61-141.26) | 71.75 (68.66-140.41) | -0.056 | 0.956 |
| FS | 10.84 (9.27-12.4) | 11.68 (9.59-13.78) | -0.690 | 0.499 | 10.45 (9.22- 11.68) | 12.41 (9.82- 15.00) | | 0.100 | 0.921 | 10.9 (9.68-12.11) | 11.33 (9.27-13.38) | -0.388 | 0.704 |
| Bi-N | 17.21 (16.03-18.38) | 17.46 (14.84-32.3) | -0.224 | 0.825 | 16.91 (15.55- 18.27) | 18.00 (15.96- 33.96) | | 0.001 | 0.999 | 18.01 (16.1-34.11) | 16.71 (15.4-32.11) | 1.241 | 0.230 |
| S | 13.51 (11.59-15.43) | 14.49 (11.37-17.62) | -0.610 | 0.549 | 12.83 (11.26- 14.39) | 15.76 (12.46- 19.07) | | -0.202 | 0.842 | 13.56 (11.87-15.24) | 14.1 (11.45-16.74) | -0.347 | 0.732 |

Note: P values result from Student *t* test. G0, G1 and G2: different numbers of galactoses, F: fucose, bi-N: bisecting N-acetylglucosamine and S: sialic acid.

**Model Parameters**

Hyperparameters:

loss:CrossEntropyLoss, k:5, epoch:300, lr:0.03, weight_decay:0.0001, batch_size:5

Features:

'IgG1 G0-NF', 'IgG1 G2NF', 'IgG2 G0N', 'IgG2 G1N', 'IgG2 G2N', 'IgG2 G2NF', 'IgG3 or 4 G0'

Network Structure:

Sequential(

(0): Linear(in_features=7, out_features=6, bias=True)

(1): Sigmoid()

(2): Linear(in_features=6, out_features=4, bias=True)

(3): Sigmoid()

(4): Linear(in_features=4, out_features=2, bias=True)

)

Network Parameters by Layers:

0.weight

[tensor([ 1.8851, 0.2173, 0.4247, 2.5979, 0.8736, 1.7704, -2.4178],

grad_fn=<SelectBackward>), tensor([ 1.8873, 0.2646, 0.4643, 2.5947, 0.8887, 1.7859, -2.4643],

grad_fn=<SelectBackward>), tensor([ 1.8859, 0.2324, 0.4388, 2.5967, 0.8781, 1.7746, -2.4334],

grad_fn=<SelectBackward>), tensor([ 1.8847, 0.2086, 0.4180, 2.5986, 0.8708, 1.7673, -2.4097],

grad_fn=<SelectBackward>), tensor([ 1.8848, 0.2233, 0.4328, 2.5973, 0.8749, 1.7708, -2.4256],

grad_fn=<SelectBackward>), tensor([ 1.8820, 0.2036, 0.4234, 2.5990, 0.8679, 1.7616, -2.4117],

grad_fn=<SelectBackward>)]

0.bias

[tensor(-4.5620, grad_fn=<SelectBackward>), tensor(-4.5929, grad_fn=<SelectBackward>), tensor(-4.5715, grad_fn=<SelectBackward>), tensor(-4.5564, grad_fn=<SelectBackward>), tensor(-4.5652, grad_fn=<SelectBackward>), tensor(-4.5511, grad_fn=<SelectBackward>)]

2.weight

[tensor([-1.4414, -1.4601, -1.4517, -1.4401, -1.4473, -1.4420],

grad_fn=<SelectBackward>), tensor([-1.7418, -1.7855, -1.7681, -1.7397, -1.7532, -1.7446],

grad_fn=<SelectBackward>), tensor([-1.7406, -1.7863, -1.7571, -1.7367, -1.7466, -1.7368],

grad_fn=<SelectBackward>), tensor([-1.7765, -1.8210, -1.7826, -1.7602, -1.7813, -1.7603],

grad_fn=<SelectBackward>)]

2.bias

[tensor(2.9029, grad_fn=<SelectBackward>), tensor(3.4966, grad_fn=<SelectBackward>), tensor(3.4876, grad_fn=<SelectBackward>), tensor(3.5417, grad_fn=<SelectBackward>)]

4.weight

[tensor([0.7949, 1.2808, 1.2813, 1.3253], grad_fn=<SelectBackward>), tensor([-0.7925, -1.2749, -1.2876, -1.3403], grad_fn=<SelectBackward>)]

4.bias

[tensor(-3.1794, grad_fn=<SelectBackward>), tensor(3.1830, grad_fn=<SelectBackward>)]
